# Supplementary material for: Learning to Centralize Dual-Arm Assembly
Source: Front Robot AI. 2022 Mar 23;9:830007. doi: 10.3389/frobt.2022.830007 (PMC8984145; doi:10.3389/frobt.2022.830007)
Supplement: Supplementary file 1 [file DataSheet1.PDF]

Supplementary Material

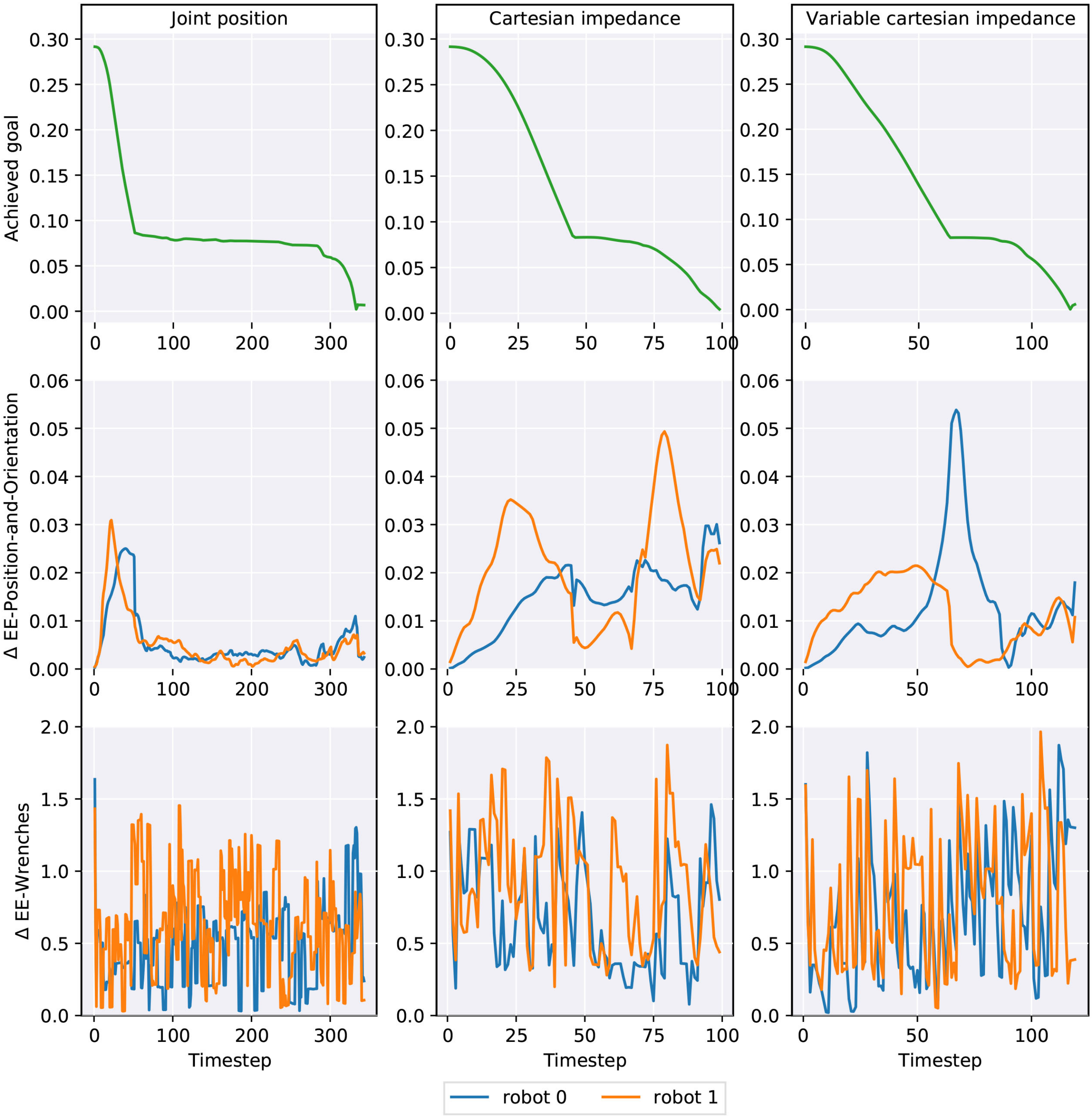

Figure S1. Example trajectories for joint position, cartesian impedance and variable cartesian impedance control. The top row shows the achieved goals, the middle row the sum of normalized changes of end-effector positions and orientations ($\sum(|pos_t - pos_{t+1}|) + \sum(|ori_t - ori_{t+1}|)$) and the bottom row the sum of normalized changes of end-effector wrenches ($\sum(|wrench_t - wrench_{t+1}|)$.

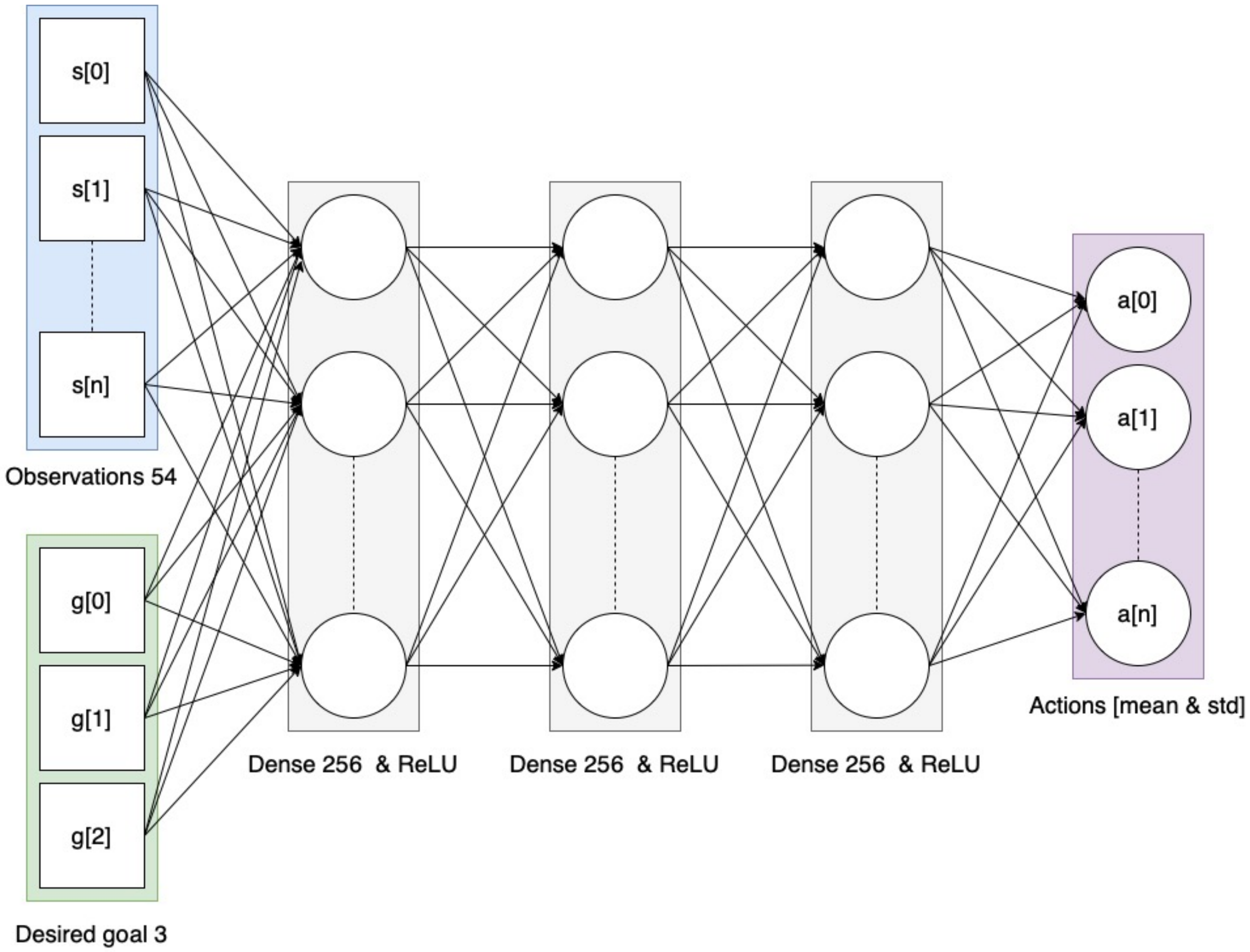

Figure S2. Policy network: The policy takes observations and desired goal as input, and outputs mean and standard deviation of actions.

Table S1. Controller implementation details for simulation and real-world.

	Joint position	Cartesian impedance	Variable cartesian impedance
Simulation	$kp = 200$	$kp = 200$ $dx_{max} = 0.2$	$kp_{min} = 10$ $kp_{max} = 400$ $dx_{max} = 0.2$
Real-world	$kp = 20$	$kp = 25$ $dx_{max} = 0.2$	$kp_{min} = 5$ $kp_{max} = 50$ $dx_{max} = 0.2$

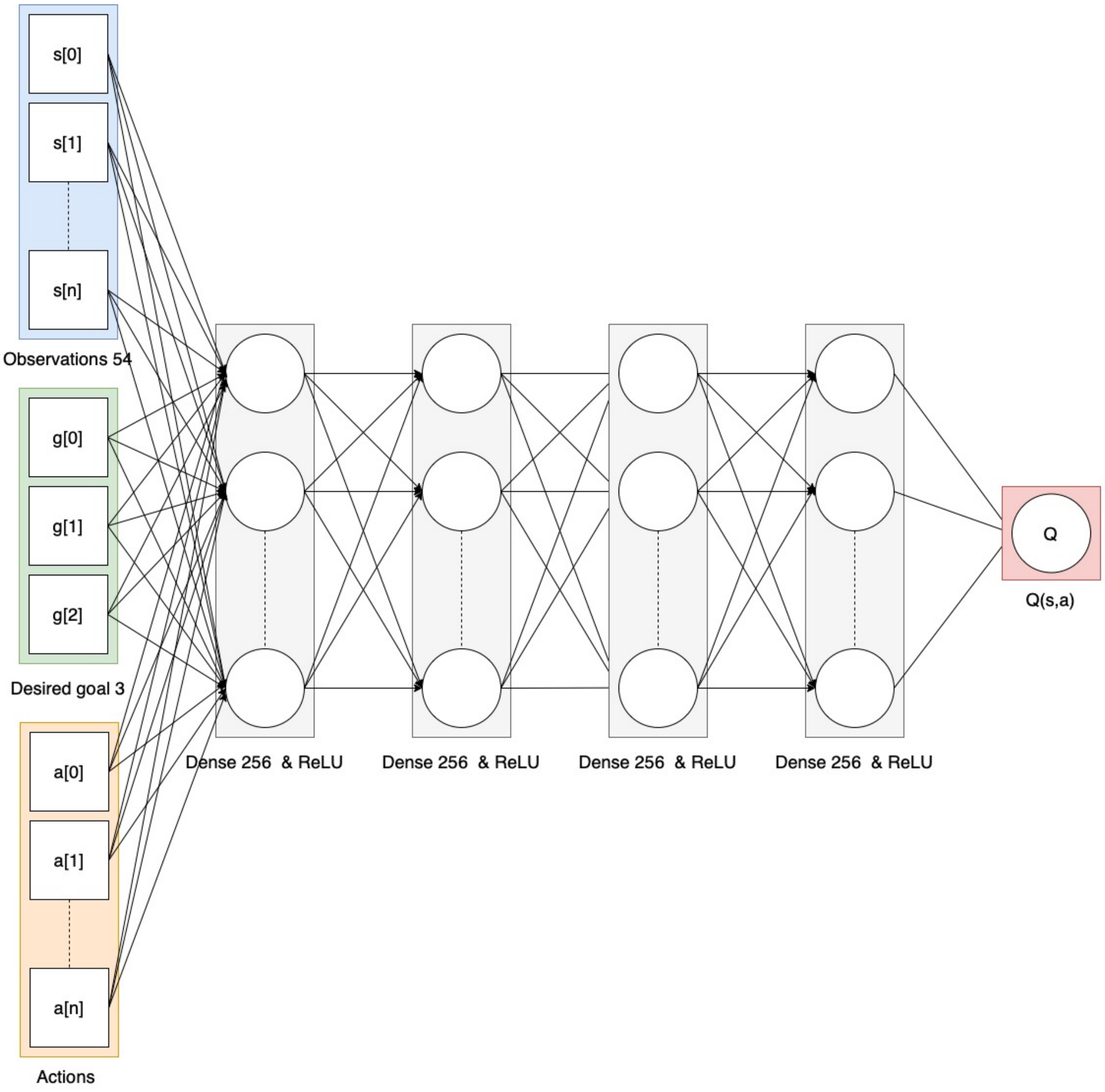

Figure S3. Q-network: The Q-network takes observations, desired goal and actions as input, and outputs the corresponding Q-value.

Table S2. Joint space limits for simulation and real-world.

	Joint space limits
Simulation	$q_{min} = [-2.7, -1.6, -2.7, -3.0, -2.7, 0.2, -2.7]\ rad$<br>$q_{max} = [2.7, 1.6, 2.7, -0.2, 2.7, 3.6, 2.7]\ rad$<br>$dq_{max} = [2.175, 2.175, 2.175, 2.175, 2.61, 2.61, 2.61]\ rad/s$<br>$\tau_{max} = [85, 85, 85, 85, 10, 10, 10]\ Nm$<br>$d\tau_{max} = 1000\ Nm/s$
Real-world	$q_{min} = [-2.7, -1.6, -2.7, -3.0, -2.7, 0.2, -2.7]\ rad$<br>$q_{max} = [2.7, 1.6, 2.7, -0.2, 2.7, 3.6, 2.7]\ rad$<br>$dq_{max} = 0.1 * [2.175, 2.175, 2.175, 2.175, 2.61, 2.61, 2.61]\ rad/s$<br>$\tau_{max} = [6, 6, 6, 6, 1.5, 1.5, 1.5]\ Nm$<br>$d\tau_{max} = 1000\ Nm/s$
